# Editorial: Phage-bacteria interplay: Future therapeutic approaches against antibiotic resistant bacteria

**DOI:** 10.3389/fcimb.2022.1124187

**Published:** 2022-12-30

**Authors:** Cecilia A. Silva-Valenzuela, Roberto C. Molina-Quiroz, Sanna Sillankorva

**Affiliations:** ^1^ Microbes Lab SpA Valdivia, Los Ríos, Chile; ^2^ Stuart B. Levy Center for Integrated Management of Antimicrobial Resistance (Levy CIMAR), Tufts Medical Center and Tufts University, Boston, United States; ^3^ International Iberian Nanotechnology Laboratory (INL), Braga, Portugal

**Keywords:** antimicrobial resistance, phage (bacteriophage), infections, coevolution, arms-race

The antibiotic resistance crisis is a worldwide healthcare concern. World Health Organization (WHO) has projected that infections generated by multi-drug resistant (MDR) bacteria will most likely increase in the near future. This situation has encouraged scientists to develop and/or improve complementary strategies to fight this threat. In this context, bacterial viruses (bacteriophages or phages) have been proposed as a therapeutic alternative due to their specificity and different mechanism of action compared to antibiotics.

Phages are the most abundant microbial entity in the environment (reaching ~10 e31), and the constant interplay with their bacterial hosts shapes both phage and bacterial evolution in the so-called arms race.

This Research Topic aimed to provide an update on the impact of phage-host interactions in bacterial evolution and the cautions to consider for administering phage-based therapies. In this context, new research showing relevant aspects of phage biology during the interaction with their hosts, such as the effect of genotypic diversity and its impact on bacterial coevolution (Castledine et al.) and the dynamics of phage-bacteria coevolution in culture media was reported (Barron-Montenegro et al.). In addition, the use of CRISPR-Cas genetic scars in the host genome as a tool to understand phage-host interplay in the human microbiome (Monshizadeh et al.) and the characterization of novel phages able to infect and kill clinically relevant MDR pathogens was also highlighted (Li et al.). Altogether, these new findings increase our current understanding of phage-bacteria interactions and contribute to reinforcing the idea of a rational use of phage therapy and the identification of novel relevant aspects to consider when using phages as an antibacterial strategy for the treatment of MDR human and foodborne pathogens.

## Author contributions

All authors listed have made a substantial, direct, and intellectual contribution to the work and approved it for publication.

